# A Review on Membrane Technology and Chemical Surface Modification for the Oily Wastewater Treatment

**DOI:** 10.3390/ma13020493

**Published:** 2020-01-20

**Authors:** Fatma Yalcinkaya, Evren Boyraz, Jiri Maryska, Klara Kucerova

**Affiliations:** 1 Centre for Nanomaterials, Advanced Technology and Innovation, Department of Nanomaterials and Informatics, Technical University of Liberec, Studentska 1402/2, 46117 Liberec, Czech Republic; evrenboyraz@gmail.com (E.B.); jiri.maryska@tul.cz (J.M.); klara.kucerova2@tul.cz (K.K.); 2 Faculty of Mechatronics, Institute for New Technologies and Applied Informatics, Technical University of Liberec, Studentska 1402/2, 46117 Liberec, Czech Republic

**Keywords:** oil separation, nanomaterial, membrane, self-cleaning, surface modification

## Abstract

Cleaning of wastewater for the environment is an emerging issue for the living organism. The separation of oily wastewater, especially emulsified mixtures, is quite challenged due to a large amount of wastewater produced in daily life. In this review, the membrane technology for oily wastewater treatment is presented. In the first part, the global membrane market, the oil spill accidents and their results are discussed. In the second and third parts, the source of oily wastewater and conventional treatment methods are represented. Among all methods, membrane technology is considered the most efficient method in terms of high separation performance and easy to operation process. In the fourth part, we provide an overview of membrane technology, fouling problem, and how to improve the self-cleaning surface using functional groups for effectively treating oily wastewater. The recent development of surface-modified membranes for oily wastewater separation is investigated. It is believed that this review will promote understanding of membrane technology and the development of surface modification strategies for anti-fouling membranes.

## 1. Introduction

The fast-growing population and industry have increased the demand for clean water. Even though 70% of the world surrounded by water, only 2.5% of it is fresh, and only 1% of the freshwater is accessible, which is shared among the 7.6 billion of its inhabitants. According to the United Nations report, the population is expected to reach 8 billion by 2030 and 9.7 billion in 2050 [1]. Currently, more than two billion people are not able to get clean water in their homes. It is unavoidable that two-thirds of the world’s population living in water-stressed regions in the near future. Deaths and diseases are increased worldwide due to water contamination. 

The demand for better water treatment technology is growing and expanding due to the effects of environmental degradation on the economy. The growth in demand increases the overall demand for membrane separation technology. The membrane separation technology divided into four processes as; microfiltration, ultrafiltration, nanofiltration, and reverse osmosis. 

From 2018 to 2023, the global membrane microfiltration market expected to reach $3.7 billion from $2.4 to 9.0% at a compound annual growth rate (CAGR) [2]. The ultrafiltration, nanofiltration, and reverse osmosis (RO) membrane markets expected to reach $2920 Million, $845.2 Million, and $12.15 billion, rising at a market growth at CAGR of 3.6%, 5.3%, and 8.7% through 2025, respectively [3,4,5]. The membrane manufacturers increase accordingly to the application area. The top manufacturers, membrane types and end users of membrane market are given in Table 1.

Every day 5 m^3^ of wastewater/person is produced in the food industry in Europe, and 250 km^3^ of water is lost per year worldwide. Almost 45,000 km^2^ marine ecosystem, fishing, livelihoods, and food chains on the seas and oceans are affected by untreated wastewater [12]. 

The recycling of wastewater has been brought to the agenda due to the speed growing in the ratio of industry and population, increase in water wages, and difficulties in the water supply. The benefits of recycling wastewater can be listed as follows.

- A reliable source of water under reliable controls and conditions.
- The demand for energy is less.
- It helps to prevent the deterioration of surface water quality.
- It leads to a reduction in the consumption of water resources.

The recovered wastewater can be used in several applications such as industrial process water, agricultural irrigation, garden and park irrigation, and artificial feeding of groundwater. 

There are several factors that fasten water contamination besides the population such as pollution. The pollutant may be formed from different sources like synthetic, heavy metals, plastics, groundwater, sediment, chemical, agricultural, pathogenic, atmospheric pollutants, pesticides and herbicides, saltwater intrusion, and oil contamination. 

The increased industrial oily waste, oil spill accidents, oily effluent discharges, and oil leakage and have become one of the top environmental concerns for human life. Waste oils are considered to possess harmful, carcinogenic and ecotoxic hazards. The extremely toxic hydrocarbons and polyaromatic hydrocarbons are the components of petroleum and wasted oil that has brought fatal damage to the environment. There have been several oil spill accidents in history. Oil spills produce ecological disasters, and can negatively influence the physiology, immunology, and development of some organisms. Some of the important are listed as follows.

June 1979, Ixtoc I oil spill accident in the southwestern Gulf of Mexico by the oil company Pemex happened. Around 140 million gallons of oil were spilled out per day [13].

July 1979, Atlantic Empress and Aegean Captain supertankers collided with each other in the Caribbean Sea, and each vessel was carrying over 200,000 tons of crude oil. Approximately 287,000 tons of oil spilled from the Atlantic Empress [14].

March 1983, a tanker hit the Nowruz Field platform in the Persian Gulf and caused an oil spill accident. Almost 80 million gallons of oil a day were flowed into the Persian Gulf [15].

In March 1989, the Exxon Valdez oil spill accident in Alaska’s Prince William Sound happened, causing 11 million gallons of oil to spill into the water [16].

At the beginning of January 1991, a vast amount of oil began to spill into the Persian Gulf had been caused by the United States’ sinking of two oil tankers. Over 240 million gallons of crude oil spilled into the Persian Gulf [17].

In May 1991, Liberian oil tanker ABT Summer exploded off the coast of Angola with a cargo of 260,000 tons of Iranian heavy crude oil; 1.9 million barrels of the laded oil cargo started to spill and spread onto the water surface [18].

In April 2010, a big oil spill accident was happened in the Gulf of Mexico due to the Deepwater Horizon drilling rig explosion. Over 60,000 barrels of oil per day were discharged. This is the largest oil spill disaster in the history of the petroleum industry [19]. Over 82,000 birds, 6000 sea turtles, 25,900 marine mammals, and tens of thousands of fish were killed by oil spill accidents according to the Centre for Biological Diversity.

The oil–water emulsion from emitted into the soil domestic wastewater is one of the most severe issues that threaten human life and ecology system. Based on this fact, an effective separation system is needed for the oil–water emulsion that has low fouling properties and is easy to apply and manage. 

The estimated global value for the cleaning of water is ~59$ billion which is expected to increase over the next eight years [20]. Therefore the oily wastewater separation is essential and valuable. The strict regulations and increased environmental awareness direct the researchers and industry to find new methods to separate oils from domestic and industrial wastewater, sea and ocean water, and oil spill mixtures. 

In this review, the categorization of oily waste and treatment of oily wastewater using various technologies will be discussed. The membrane technology is currently one of the most used methods in the separation of an emulsified oil–water mixture. Besides the enormous advantages of using membranes, membrane fouling is one of the most significant disadvantages of separation technology. To the best of our knowledge, many good papers focus on the development of hybrid membranes for the separation of oily wastewater [21,22,23,24,25,26]. Although, there is plenty of literature exists on the chemical modification of membranes [27,28,29,30], there are still challenges to develop a reliable method with high flux, selectivity, and self-cleaning properties. Considering the needs in this area, the surface-modified membranes are reviewed in this contribution aiming to highlight this exciting technique and provide new insight into oil–water separation.

## 2. Oily Wastewater

The oily wastewater pollution causes several problems: (a) Affecting water sources, drinking water, etc., (b) endangering human health, (c) pollution of the atmosphere, (d) affecting agriculture production, (e) harming nature, and (f) endangering the life of living organisms [31,32,33,34,35]. After many industrial processes (such as food, ship, oil refinery, petrochemical, leather, and metal finishing), oily wastewater is produced. It is necessary to clean the oils and greases (FOGs) from the water before reusing the water or discharged into sewer systems and to the surface waters. The oil is in the form of an emulsion in the enterprises is trying to comply with the discharge limits. The range of discharging limit for synthetic and mineral oils and grease is 10–15 mg/L, and those of animal and vegetable origin is 100–150 mg/L [36].

The source of the oil can be animal, vegetable, or mineral. The content of the oil can be categorized according to their physical form [36]:

(1) Free (floating) Oil: it arises quickly to the surface of the water under settled conditions. The droplet size is more than 150 microns.
(2) Dispersed oil: Electrically charged fine droplets surfactants stabilized. The droplet size is between 20–150 microns. Dispersed oil consists of polyaromatic hydrocarbons and some alkyl phenols that are less soluble in water [37].
(3) Emulsified oil: Even though the distribution is similar to dispersed oil, it is more stable due to the use of surfactants. The droplet size is smaller than 20 microns.
(4) Dissolved oil: Water-soluble oil, which is translucent and transparent. The droplet size is smaller than 5 microns.

It is necessary to use a proper treatment method or combined methods to clean oil wastewater. Based on the physical form, these methods can be show differences. In the next section, the treatment method is introduced with its pros and cons.

## 3. Common Oily Wastewater Treatment Methods

Oil pollution has long-term damage effects on the environment, risk on health and loss of energy. Various methods have been developed to separate oily wastewater, such as flotation, coagulation, biological treatment, adsorption, membrane separation, and so on. However, many of these technologies suffer from low separation efficiency, high energy cost, long-term operation, and secondary pollution. In the following section, each method is explained shortly.

### 3.1. Flotation

In 1969, the industrial use of air flotation devices for oily wastewater separation had begun [38]. Flotation processes include dissolved air flotation, induced air flotation, nozzle air flotation, and electroflotation. In this process, a gas bubble is needed to collide with and attach to oil droplets. The oil droplets attach to the bubble and rise rapidly through the water. The density difference between the floating oil and water keeps water in the bottom, and the scum layer is separated from the water. The advantages of this method are producing less sludge and separation efficiency. There is a huge potential to treat oily wastewater using the flotation method. However, there are some disadvantages such as repairment, device manufacturing, high energy consumption, generation of a large amount of air, the retention time for separation, and skim volume [39].

### 3.2. Coagulation

In the coagulation method, the surface charge of the droplets and the separation of the oil droplets reduced by coagulants, which promote the dispersion of the emulsion. This method is followed by the separation of the aqueous and oily phases by conventional precipitation or dissolved air flotation. 

Coagulation has many disadvantages, including the requirement of a high amount of coagulant, high treatment cost, long operation time, a large area of construction, corrosion problems due to the decrease in pH and very costly sludge production, increased concentration of metals in effluents, and it can cause secondary pollution [39,40].

### 3.3. Biological Treatment

Biological processes generally eliminate oils and fats using biological degradation and are less expensive than chemical equivalents. In this method, dissolution of water is done by microbial metabolism and colloidal organic pollutants, which are transformed into stable harmless substances. The activated sludge and biological filters are commonly used. The activated sludge in the aeration tanks is concentrated on the surface of the microorganisms, which hold on to the filter to separate the organic matter, using the current state vector as adsorption purifying microorganisms. This method has the potential for the treatment of large-scale heavy petroleum wastewater [31]. The disadvantages are high consumption of oxygen, efficacy decreases as the concentration of pollutant increases, energy-intensive, requires a qualified operator, and elevated operating costs [37]. Moreover, some of the organic compounds are resistant to biological cleanup [40]. 

### 3.4. Adsorption

In the adsorption method, the pollutant chemicals are attached to the surface of a solid by physical adhesion. Using this method, most of the pollutants can be removed, and almost all of the wastewater can be recovered. Activated fishbone charcoal (MAFC) for the separation of emulsified oil from oily wastewater has been prepared [41]. Potassium carbonate (K_2_CO_3_) is used as an activating agent and adsorption performance for removing emulsified oil has been observed. The maximum removal rate of emulsified oil reaches 90.1%. However, in each recycling, the adsorption capacity decreased drastically. Similar results have been reported in [42,43,44]. Even though the adsorption method has been widely used in the treatment of oily wastewater, the adsorbents have to replace after a relatively short period of operation or need to be removed. This is due to the saturation of the adsorbents with a high concentration of waste. The replacement and regeneration are costly and unfavorable for long-term operation.

### 3.5. Membrane Technology

Membrane technology is a more efficient method than the conventional separation methods. A membrane is a barrier between two phases, which separates and limits the transport of many chemicals selectively. Based on their structure, membranes can be grouped into four categories: homogeneous, heterogeneous, symmetric, or asymmetric. Membranes can separate solid or liquid and also can carry a positive or negative charge or be neutral or bipolar. The membrane does double duty for separation, and the separation is straightforward. First, the membrane behaves like the semi-permeable layer between two phases and second transports between two phases. The efficiency of the membranes is dependent on the membrane itself.

As an effective method, membrane technology is one of the most commonly used methods for the separation of oil–water wastewater or emulsions, in food processing, pharmaceutical, desalination, and fuel cell industries. In comparison to other treatments, the membrane separation method has higher efficiency, consistent effluent quality, and lower consumption of energy [45]. Based on the separation and pore size type, membranes can be grouped as microfiltration (MF, pore size ranges from 0.1–5 µm), ultrafiltration (UF, pore size ranges of 0.01–0.1 µm), nanofiltration (NF, pore size range of 0.001 to 0.01 µm), and reverse osmosis (RO, pore size range of 0.0001 to 0.001 µm) which are the pressure-driven processes [46,47]. The main types of membrane are classified as follows.

- Isotropic membranes: (a) microporous membranes, (b) nonporous, dense membranes, and (c) electrically charged membranes
- Anisotropic membranes
- Ceramic, metal, and liquid membranes

The membrane technology is suitable for the treatment of oily wastewater and is more useful and promising than traditional methods to remove oil droplets (especially below 10 µm) and more efficient. 

## 4. Membrane Fouling and Surface Modification

The membrane fouling can be defined as the “process resulting in loss of performance of a membrane due to deposition of suspended or dissolved substances on its external surfaces, at its pore openings, or within its pores”, which results in deterioration of the membrane [48]. 

The main reasons for the membrane fouling are [49]

- deposition of sludge flocs or particles on the membrane surface,
- adsorption of solutes or colloids within/on membranes, and
- formation of cake layer on the membrane surface.

Membrane fouling not only reduces water permeability and separation efficiency, but also reduces the membrane life-span, productivity, and permeate quality, while also increasing operation cost as well as a reduction in membrane lifetime [50]. Zoubeik et al. [51] divided the flux decline of the membrane into three stages. At the first stage, the decline of flux is very sharp which shows the rate of fouling is the highest. In the second stage, the fouling rate is slow down as the decline of flux becomes more gradual. In the third stage, the fouling rate becomes zero and flux is in the steady-state form under the constant transmembrane pressure.

Adsorption of organic molecules can cause the membrane fouling. In the oily wastewater separation process, decreasing the adsorption of oil droplets and organic contaminants on the membrane surface is one of the most critical issues. The oil droplets and organic contaminants cause the membranes to be polluted and blocked. To remove these contaminations can reduce membrane life. For this reason, improved antifouling performance and efficiency of the membranes are desirable.

To avoid the fouling and optimize the hydrodynamic conditions of the membrane, different methods have been applied. Surface modification is one of the effective methods that can improve membrane antifouling. Using a surface modification system, one can increase the hydrophilicity of the membrane and also reduces organic foulant adsorption on the membrane surface [52,53]. Hydrophilic membranes tend to antifoul during filtration [54,55].

Nowadays, the hydrophobic membranes can be changed to the strongly hydrophilic membranes by applying Al_3_O_4_, SiO_2_, Fe_3_O_4_, ZrO_2_, and TiO_2_ inorganic nanoparticles into the membrane. Because of its photocatalytic and super-hydrophilic effects, TiO_2_ is prevalently used for membrane modification [56]. The surface coating is by self-assembly of TiO_2_ particles via coordinance bonds with OH functional groups of polymer on the membrane do not create only photocatalytic property but also increases the hydrophilicity of the membrane [54,57].

The main duty of the surface modification is to improve membrane hydrophilicity which improves the membrane performance. Hydrophilic polymers such as poly(ethylene glycol), poly(ethylene glycol) methyl ether methacrylate, poly(2-hydroxyethyl methacrylate), poly(acrylic acid), or zwitterionic polyelectrolyte were chemically modified onto membrane surfaces via the formation of covalent bonds. The results indicated that the hydrophilic membranes form compact hydrated layers to prevent fouling of the oil droplets on the membrane surfaces and to make the easy oil removal during cleaning process [58,59,60,61].

Yu et al. [62] fabricated corn straw powder (CSP)-nylon 6,6 membrane (CSPNM) by a phase inversion process without any further chemical modification. The resultant membrane showed superhydrophilic and underwater superoleophobic characteristics. CSP significantly increased the mechanical strength, oil rejection (over than 99%), and flux (over 660.00 L/(m^2^h)) of the membrane. After 20 cycles of separation, the oil rejection and flux have no visible change with the good antifouling property.

**Figure 1:**
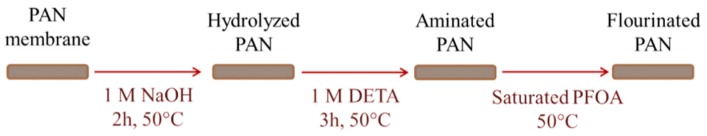
Schematic illustration of surface modification of PAN membrane (DETA = Diethylenetriamine, inspired from work in [63]).

Fluorinated polyacrylonitrile (PAN) membrane is prepared by grafting a low surface free energy molecule pentadecafluorooctanoic acid onto aminated PAN membrane surface through the acylation reaction between the carbonyl groups of pentadecafluorooctanoic acid (PFOA) and amine groups on aminated PAN membranes as shown in Figure 1. Grafting of perfluoroalkyl groups onto the aminated PAN membrane surfaces manipulated the physicochemical features; and as a result, an excellent antifouling property was obtained. The membrane surface energy significantly lowered by the presence of non-polar hydrophobic perfluoroalkyl groups on the membrane surfaces [63].

**Figure 2:**
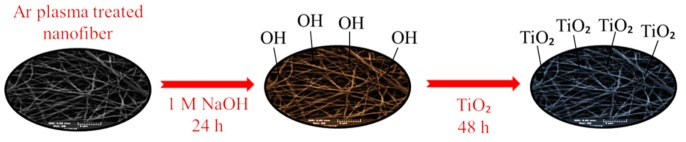
Schematic illustration of surface modification of PVDF/PAN nanofibrous membrane (inspired from work in [54]).

Hydrophilic/oleophobic PVDF/PAN nanofibrous hybrid membrane was prepared using a two-step modification (illustrated in Figure 2). In the first step, hydrophilic −OH groups were introduced onto the membrane surface using low-vacuum microwave argon plasma treatment, followed by sodium hydroxide (NaOH) immersion. In the second step, titanium dioxide (TiO_2_) nanoparticles were synthesized and grafted onto PVDF/PAN-OH membrane surface. The resultant membranes showed enormous water permeability (over than 160,000 (L/m^2^hbar)) with excellent antifouling property [54].

**Figure 3:**
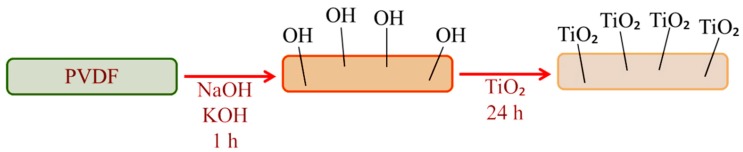
Schematic illustration of surface modification of PVDF nanofibrous membrane (inspired from [64]).

A similar attempt has been made using PVDF nanofiber web. Differently, the plasma treatment has been eliminated [64]. PVDF membranes were defluorinated in alkaline solution, and then TiO_2_ nanoparticles were attached on the surface, as illustrated in Figure 3. Results indicated that after adding TiO_2_ nanoparticles, membranes exhibited outstanding antifouling and self-cleaning performance with high selectivity.

**Figure 4:**
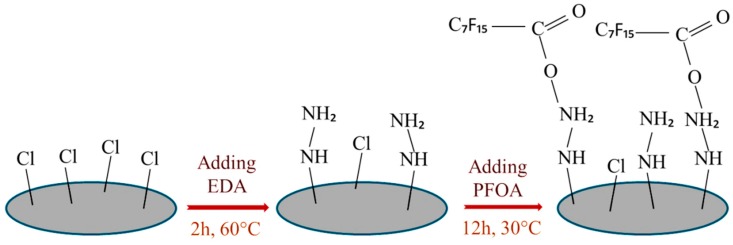
Schematic illustration of surface modification of PVC fluorinated membrane (inspired from [65]).

Novel fluorinated membranes were fabricated by polyvinyl chloride (PVC), and chlorinated polyvinyl chloride (CPVC) blend membranes. First, ethylenediamine (EDA) was used for the amination of PVC/CPVC blend membranes by chemical grafting method. Second, by using the chemical reaction between the redundant amino group of EDA and the carboxyl group of pentadecafluorooctanoica acid (PFOA), fluorination treatment was achieved (illustrated in Figure 4). The fluorinated membranes exhibited high permeate fluxes (over 40 L/(m^2^h)), low flux decline, and high flux recovery in oil/water emulsion [65].

**Figure 5:**
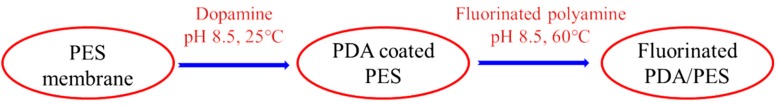
Schematic illustration of preparation and surface fluorination for the fluorinated PDA/PES membrane (inspired from work in [66]).

Antifouling and high-flux (~46.1 L/(m^2^h)) polydopamine (PDA) membranes were developed using chemical modification by Michael’s addition reaction between fluorinated polyamine and quinone groups of PDA [66], as shown in Figure 5. A thin PDA layer deposited onto the polyethersulfone (PES) membrane surface and the modification took place. The new membrane exhibited excellent antifouling properties and the flux recovery rate (~98.6%).

Membrane modification shows a significant effect on membrane flux and permeability. Moreover, some of the membranes improve self-cleaning properties. Literature research shows that, for effective and high-performance oil separation, surface modification of membrane is needed [58,59,60,61,62,63,64,65,66]. Surface modified membranes not only offer superior flux but also antifouling performance. This technology opens a new direction for the membrane design with improved performance and self-cleaning surface. A sustained effort and extensive study should be performed to improve surface modification stability.

## 5. Membrane Cleaning

The major obstacle for the application of membrane processes is the membrane fouling. The problem is characterized by an irreversible decline in the flux over time and an increase in hydraulic resistance, due to interactions with various components in the feed solution. During the mass transport, the particulate materials can attach, accumulate, or adsorbed onto membrane surfaces and/or within membrane pores. The adsorption of organic pollutants on the membrane surface causes severe fouling because of hydrophobic nature and low surface energy of materials. The membrane quality is affected by membrane fouling. 

The fouling can be classified into two groups as reversible and irreversible which is due to chemisorption and pore plugging mechanisms. In the reversible fouling, a cake layer formation or concentration polarization of materials is observed on the membrane surface. The reverse fouling can be back-washable or non-back washable. The back-washable fouling can be cleaned by backwashing or surface washing. On the other hand, the non-back-washable fouling can be cleaned only by extensive chemical cleaning which possibly increases the cost and decreases the membrane life. In the chemical cleaning, chemicals such as nitric acid (HNO_3_) and hydrogen chloride (HCl), or disinfectant (such as hydrogen peroxide (H_2_O_2_)) are added to the permeate during the backward flush. When the membrane used for a long term, the fouling is not reversible. The number of filtration cycles increases the irreversible membrane fouling. Therefore, it is good to improve the hydrophilicity of the membrane to decrease fouling and increase the flux of membranes. 

Oxidation processes have great potential in the degradation of organic pollutants. The organic pollutants attached to the membrane surface can be degraded under visible or UV light irradiation, which improves the self-cleaning property of the membranes. Anderson et al. studied the possibility of binding of photocatalytic TiO_2_ functionalization with membrane separation [67]. Later, the method of research triggered the preparation of TiO_2_-embedded membranes [68,69]. For example, Damodar et al. used blending different amounts of TiO_2_ and prepared modified polyvinylidene difluoride (PVDF) membranes. Moreover, they investigated antibacterial, photocatalytic, and antifouling properties of the membrane. As a result of the additional TiO_2_, it was found that the pore size and hydrophilicity of the membrane were affected by additional TiO_2_ the water permeability of the PVDF/TiO2 membrane increased. Oxidation method is one of the used methods in the industry for membrane cleaning. Additionally, under UV light exposure, PVDF/TiO_2_ membranes exhibited anti-bio and -organic fouling abilities [70]. Xie et al. [71] developed photo-Fenton self-cleaning PVDF/TA/β-FeOOH membranes by green tannic acid (TA-Fe(III)) complexes assembly and followed in situ mineralization of β-FeOOH. Results indicated that the β-FeOOH with robust photo-Fenton catalytic activity removed oil foulants adsorbed onto the membrane surface by catalytic degradation. Membranes with super hydrophilicity/underwater superoleophobicity showed high efficiency and flux for the separation of oily wastewater. The antibacterial efficiency of the membrane is based on the hydroxyl radicals produced by UV radiation. Moreover, the UV radiation can destroy bacterial DNA and inhibit bacterial growth. However, UV treatment is highly costly and limited in applicability.

Chlorination is one of the most commonly used disinfection processes to prevent biofouling during water pretreatment. The aim of disinfection is to prevent the colonization of bacteria on the membrane surface. However, it has several disadvantages, such as not effectively working against some type of bacteria, the formation of disinfection by-products, and damaging of the membrane [72,73].

Yang et al. [74] coated the surface of the PVDF microfiltration membrane using Gallic acid (GA)/γ-aminopropyltriethoxysilane (APTES) to achieve high hydrophilicity. The hydrophilization was achieved to the synergistic effect with the integration of the mussel-inspired biomimetic hybrid network and the in situ biomimetic silicifications via “pyrogallol-amino covalent bridge”. The resultant membranes showed very high flux (9246 L/(m^2^h)) with an oil rejection greater than 99.5%. Using this method, the surface structure of the membrane which plays a role in hydrophilicity can be changed.

Ultrasound treatment is another alternative method to control membrane fouling. When the ultrasound (US) waves propagate in the feed solution, the liquid tensile strength is exceeded. As a result, gas bubbles form in the negative pressure waves, then grow and collapse in the positive waves. Intense localized energy is released [75]. Depends on optimal ultrasound frequency, power density, and irradiation direction, US treatment can improve filtration performance as well as the flux of the membranes.

Even though several ways have been reported for membrane cleaning, it is still a big challenge to find a way that costs less time and energy and fewer chemicals for the cleaning of the membranes. Using surface modification, self-cleaning surfaces can be prepared. In this way, the general membrane cleaning process (such as backwash or chemical cleaning) cycles can be reduced. Surface modification is not only improving the membrane anti-fouling, but also flux, permeability, and the selectivity of the membrane. However, despite these advances, research toward the surface modified membranes remains far less developed and a thorough study is highly desired.

## 6. Conclusions

Oily wastewater is found in many industries like petrochemical, textile, painting, food, metal finishing, etc. Proper treatment has to be done for the oily wastewater to protect the global risk to the environment and human health. The present review focuses on membrane technology for water treatment, especially oily wastewater. The main obstacle for the membranes is fouling which can reduce membrane performance, lifetime and increase operational cost. Antifouling membranes gain importance that providing superiority in performance, long-term durability, and high selectivity. Increasing the hydrophilicity of the membrane decreases membrane fouling. To date, many researchers focus on functional design membranes to reduce fouling. Surface modified membranes show high antifouling properties. However, the uniformity of modification across large membrane area, the cost of chemicals and the stability of the modification has to be addressed. An ideal surface modification technique will introduce desired functional groups/nanoparticles on the membrane surface with antifouling property and without producing significant hazards.

## Figures and Tables

**Table 1:** Current membrane type, manufacturers, and end users in the market.

Type of Technology	Manufacturers	Type	End Users	References
Microfiltration	Asahi Kasei Totay Mitsubishi Rayon KMS GE Water and Process Technologies Toyobo KUBOTA Sumitomo Electric Industries Evoqua X-Flow (Pentair) IMT Lenntech Synder Filtration MICRODYN-NADIR CLARCOR Industrial Air TriSep MOTIMO Origin Water Zhaojin Motian Ningbo Changqi Porous Membrane Technology RisingSun Membrane Delemil Yantai Gold Water Membrane AMFOR INC	PVDF PTFE PES Other	Industry Municipal water Wastewater Treatment Others	[6,7]
Ultrafiltration	Koch Asahi Kasei GE Water and Process Technologies Evoqua DOW Toray 3M (Membrana) Mitsubishi Rayon Nitto Denko Corporation Degremont Technologies Basf Synder Filtration Microdyn-Nadir Canpure Pentair (X-Flow) Applied Membranes CITIC Envirotech Litree Origin Water Tianjin MOTIMO Zhaojin Motian Memsino	Inorganic Membrane Organic Membrane	Food and Beverage Industrial and Municipal Healthcare and Bioengineering Seawater Reverse Osmosis Potable Water Treatment	[8,9]
Nanofiltration	The DOW Chemical Company Hydranautics (NittoDenko) Toray Industries Inc. Koch Membrane Systems Inc. Alfa Laval AB Pall Corporation GEA Filtration Hyflux Ltd. Inopor GmbH Argonide Advanced Water Filtration Systems	Polymeric Inorganic Hybrid	Food and Beverages Chemical and Petrochemical Water and Wastewater Treatment Pharmaceutical Biomedical Textiles Agriculture Others	[10]
Reverse osmosis	The Dow Chemical Company General Electric Koch Membrane Systems Toray Group Toyobo Applied Membranes NanOasis Nitto Denko Xylem PCI membranes Pure Aqua	Cellulose-based membranes Thin film composite membranes	Desalination RO Purification Systems Others	[11]
